# The impact of environmental contaminants, air pollution, and social deprivation on childhood leukemia survival in California

**DOI:** 10.3389/fonc.2025.1686115

**Published:** 2025-12-08

**Authors:** Fernando Hernandez, Eric Stewart, Libby M Morimoto, Alice Y Kang, Lena E Winestone, Catherine Metayer

**Affiliations:** 1 Division of Epidemiology, School of Public Health, University of California, Berkeley, Berkeley, CA, United States; 2 Division of Allergy, Immunology, and Blood and Marrow Transplant, Department of Pediatrics, University of California San Francisco (UCSF) Benioff Children’s Hospitals, San Francisco, CA, United States; 3 UCSF Helen Diller Family Comprehensive Cancer Center, San Francisco, CA, United States

**Keywords:** children, acute lymphoblastic leukemia, acute myeloid leukemia, CalEnviroScreen, air pollution

## Abstract

**Introduction:**

Despite advances in leukemia treatment, disadvantaged children experience worse outcomes. We evaluated the impact of intertwined socioeconomic and pollution burdens on childhood leukemia survival, focusing on ambient air pollution, a known carcinogen.

**Methods:**

Participant data were obtained from the California Childhood Leukemia Study (1995-2015) and linked by diagnosis residence to the CalEnviroScreen (CES) 3.0 database, that characterizes neighborhood pollution burden and area-based population vulnerabilities across California. Five-year survival analyses were performed using Kaplan-Meier estimators and Cox proportional hazards ratio (HR) models with 95% confidence intervals (CI), adjusting for individual socio-demographic and clinical prognostic factors.

**Results:**

124 out of 1,210 children with acute lymphoblastic leukemia (ALL) died within 5 years of diagnosis. Living in an area with a medium/high CES composite score for both pollution and population/social burdens was associated with decreased ALL survival, compared to a low CES score (HR = 2.19; 95% CI: 1.19-3.91), with population/social burden driving this observation (HR = 1.85; 95% CI: 1.06-3.21). HRs for medium/high composite, particulate matter (PM2.5), and ozone scores were the highest among children with ALL molecular subtypes known to have unfavorable prognosis (i.e., high-hyperdiploidy negative, *CDKN2A* and *IKZF1* deletions). For acute myeloid leukemia (AML) (49 deaths among 178 cases), living in an area with medium/high score for population/social burden was associated with an increased risk of death (HR = 2.21; 95% CI: 0.99-4.94).

**Conclusion:**

High cumulative community burden, especially social deprivation, was associated with reduced survival of childhood ALL and AML, while high levels of PM2.5 and ozone were associated with reduced survival in specific ALL subtypes.

## Introduction

Cancer is the leading cause of death by disease among children in the United States (US) (1). Leukemia is the most common childhood cancer in industrialized countries, comprised mainly of acute lymphoblastic leukemia (ALL), followed by acute myeloid leukemia (AML). The incidence of childhood leukemia in the US has increased since the 70’s, especially amongst the Hispanic/Latinx population (2, 3). Despite improvements in survival, children of color and those from economically disadvantaged backgrounds continue to experience greater mortality (4–10).

While differences in socioeconomic status (SES) have been associated with varying survival outcomes, disparities in treatment do not fully explain the differential clinical outcomes observed in childhood leukemia (11, 12). Exposure to pollutants is often concentrated in low SES areas, which are typically inhabited by minority groups (13), and can compound the impacts of social deprivation, worsening survival rates among those with childhood leukemia. Indeed, environmental chemical exposures may influence leukemia survival through multiple biological pathways such as increased oxidative stress, heightened inflammation, and immune dysregulation (14). These processes have been defined as drivers in leukemia as they affect immune defense-response to cancer proliferation, which in turn affects cancer risk, severity of disease, and survival (15–17). Several chemicals have been shown to increase the risk of developing childhood leukemia, yet there is little research on their impact on survival. A major component of environmental burden is air pollution that is comprised of various toxic and/or carcinogenic compounds (i.e., particulate matter (PM2.5), ozone, polycyclic aromatic hydrocarbons, etc.). Air pollution is a risk factor for cancers in adults and children (18, 19), and new evidence is emerging for its effects on childhood cancer survival (20–23). Similarly, tobacco smoke, which partly shares chemical compositions and toxicity with air pollution (24), has also been linked to reduced survival rates in childhood leukemia patients in Spain (25) and California (26).

The objective of our study was to evaluate the independent and combined effects of environmental contamination, particularly air pollution, and social deprivation on childhood leukemia survival in California. We examined pollution burden and socioeconomic deprivation and their effects on survival on both additive and synergistic scales. We hypothesize that a higher overall CES burden is associated with decreased 5-year survival among childhood leukemia patients. Additionally, we hypothesize that, after adjusting for SES deprivation, air pollution remains an independent predictor of reduced survival in childhood leukemia.

## Materials and methods

### Study population

The California Childhood Leukemia Study (CCLS) is a case-control study designed to examine environmental exposures, genetic factors, and childhood leukemia risk (1995–2015) (27); for this analysis, only ALL and AML cases were analyzed, excluding controls. Children with leukemia were rapidly identified after diagnosis across 17 hospitals in California. Eligibility criteria included age at diagnosis <15 years, English or Spanish-speaking parent(s), diagnosis residence in one of the study counties, and no previous cancer.

### Data collection

**Figure 1:**
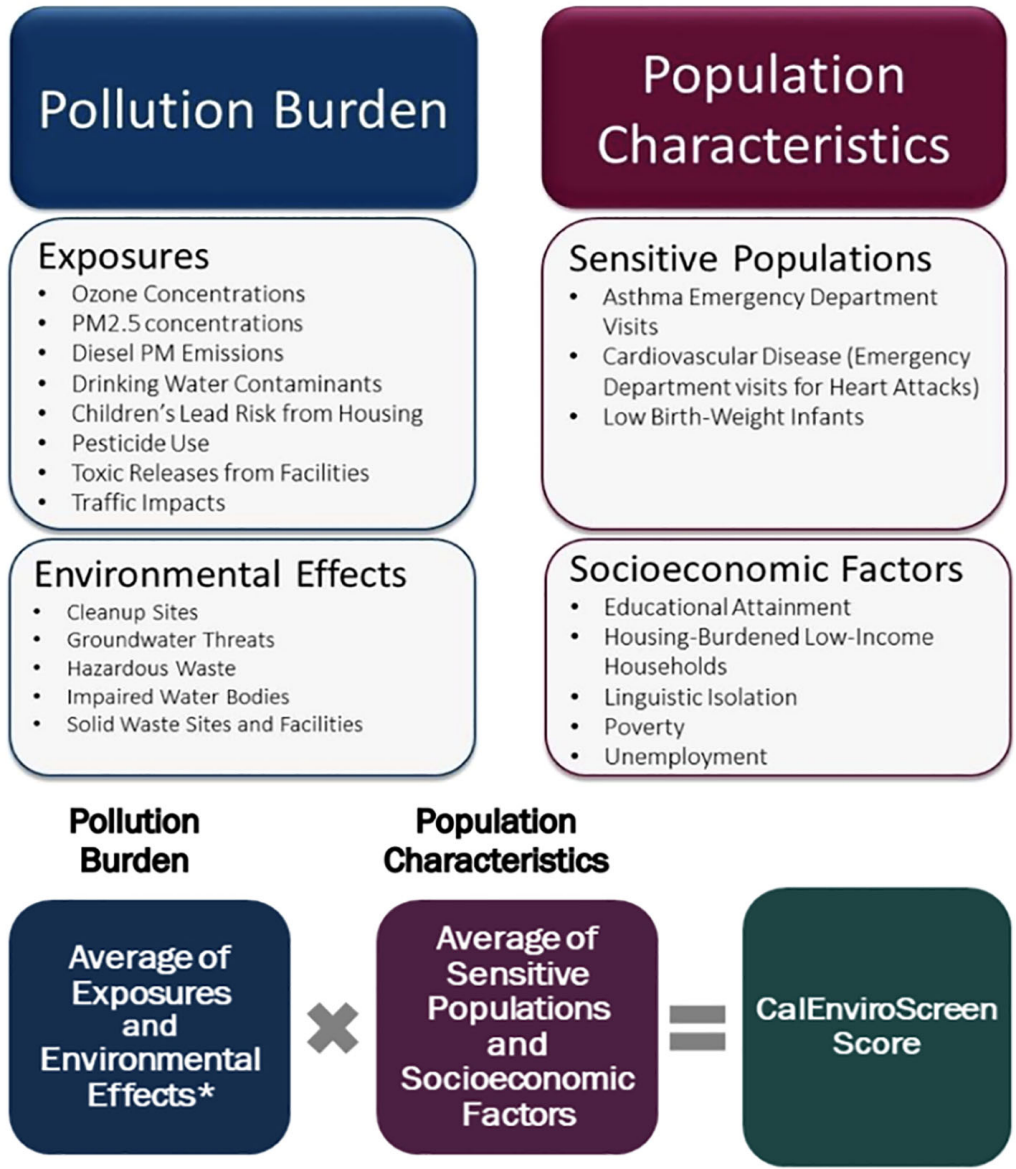
CalEnviroScreen3.0 makeup and indicators. Source: OEHHA (2017), CalEnviroScreen 3.0 Report (https://tinyurl.com/3vwc56dt).

Interviews were conducted with parents to gather information on sociodemographic characteristics, medical history, lifestyle, occupations, and residential history. Residential addresses at diagnosis were geocoded using ArcGIS Pro 3.2.0, and latitude and longitude coordinates were spatially linked to 2010 census tracts using the tigris, zipcodeR, and sf packages in R (version 4.4.0). Census tracts were linked to data from the California Communities Environmental Health Screening Tool CalEnviroScreen3.0 (CES 3.0), a publicly available database that contains data on environmental, health, and socioeconomic indicators. CES 3.0 evaluates cumulative pollution burdens and community vulnerabilities across regions in California at the census tract level (28). CES 3.0, released in 2017, was chosen because it offered the greatest temporal overlap with the study period and incorporated several important methodological improvements over earlier versions. These enhancements included the removal of less informative indicators, such as age, refinement of exposure and vulnerability metrics, and improved data quality and spatial resolution at the census tract level, making it a more robust and representative tool for assessing cumulative environmental burden and social vulnerability. The CES composite score is a cumulative measure calculated as the product of two components: the *pollution burden score* and the *population characteristics score* (**Figure 1**). The pollution burden score comprises exposures (e.g., ozone concentration, diesel PM emissions, etc.) and environmental effects (e.g., hazardous waste sites, cleanup sites, etc.). The population characteristics score considers sensitive populations (e.g., asthma-related emergency department visits, low birth weight infants, etc.) and socioeconomic factors (e.g., poverty, educational attainment, etc.). PM2.5 and ozone concentrations were used to assess ambient air pollution, given their widespread and significant health threats identified by the California Air Resource Board (CARB) (29). Annual mean PM2.5 concentrations for the years 2012 to 2014 were measured using ground-level air monitoring data from California Air Resources Board monitoring stations. Mean concentrations for each census tract were estimated using geostatistical methods, incorporating data from nearby monitoring sites. Quarterly PM2.5 data were averaged to calculate annual means, which were then averaged across the three-year period to derive the final annual mean concentrations. Ozone concentrations were obtained from CARB’s air monitoring network database for the years 2012 to 2014. During the summer months (May to October), daily maximum 8-hour ozone concentrations were calculated from ground-level monitoring sites. These values were averaged across the monitoring period to estimate mean summer concentrations. To spatially estimate ozone exposure, the mean concentrations from monitoring sites were interpolated across California census tracts using an Inverse Distance Weighting (IDW) modeling technique, producing a daily maximum 8-hour concentration (ppm) for each tract (28)]. Composite and individual CES scores were broken down into tertiles and subsequently dichotomized into low *vs*. medium/high scores (since similar results were observed with medium and high levels). This approach was chosen to optimize statistical power. Families in the medium/high exposure category for PM2.5 had an average of 12.3 µg/m^3^, above the US Environmental Protection Agency (EPA) standard of 9 µg/m^3^. For ozone, those in the medium/high category had a mean of 0.056 parts per million (ppm), which falls within the EPA’s moderate category (0.055–0.070 ppm).

Vital status was obtained by linking electronic death certificate data from the California Department of Public Health Center for Health Statistics and Informatics (1995–2020) to the CCLS database, using a probabilistic linkage (MatchPro Version 2.0.7, SEER). Inconclusive record matches (n=65) were independently reviewed by two raters to achieve consensus. Among the 1,449 participants, 191 cases were linked to death records, including 5 deaths due to external causes. Time-to-event data were constructed using each participant’s diagnosis date as the starting point, with outcomes tracked over a 5-year follow-up period. Patients who did not experience the event during this period were censored at the end of the 5-year follow-up.

Known clinical prognostic factors were collected from medical records, including histologic leukemia type and white blood count (WBC). Cytogenetic subtypes were abstracted from both medical records and additional testing including FISH to identify leukemias with high-hyperdiploidy and *TEL-AML* (*ETV6-RUNX1*) translocation, and multiplex ligation-dependent probe amplification to identify *CDKN2A* and *IKZF1* deletions (27, 30). Children without high-hyperdiploidy or with *CDKN2A* or *IKZF1* deletions, have been shown to have poorer prognosis, reducing their chance of survival (31).

Out of 1,449 cases with interviews, 1,422 provided residential addresses at diagnosis, and 1,419 were successfully linked to census tracts and matched to CES3.0 data. Of the 1,419 participants, 5 were excluded due to accidental deaths and 26 with other leukemia subtypes were excluded, resulting in a final sample of 1,388 children: 1,210 with ALL and 178 with AML (**Supplementary Figure 1**).

### Statistical analysis

The 5-year survival outcome was evaluated for all-cause mortality, excluding external causes, for ALL and AML patients. Patients were classified as an event if death occurred at any time before the end of follow-up (December 31, 2020). Patients were censored if the individual was alive at the end of follow-up or if death resulted from an external cause. The non-parametric Kaplan–Meier estimator was used to estimate the survival function, and survival curves were generated to visualize and compare the probability of survival across CES 3.0 measures. Differences in survival between groups were assessed for statistical significance using log-rank tests. Multivariable adjusted Cox proportional hazards regression was used to calculate hazard ratios (HR) and 95% confidence intervals (CI) associated with census-tract level environmental contaminants and social deprivation from CES. Covariates for the survival analysis were selected *a priori* based on their established relevance in childhood leukemia prognosis, informed by previous literature and clinical expertise. We utilized a directed acyclic graph (DAG) to conceptualize the relationships between key variables and to identify potential confounders included in the models (**Supplementary Figure 2**). Specifically, self-reported socioeconomic variables were incorporated to account for known disparities in survival outcomes. Two models were developed: one without individual-level SES adjustment and one with SES adjustment. The model without individual SES adjustment controlled for composite race and ethnicity (Latinx, non-Latinx White, non-Latinx Black, non-Latinx Asian, and non-Latinx other), birth year (continuous), and risk group (ALL only; categorical: “standard,” defined as age >1 year and age <10 years and WBC <50,000/mL; “high,” defined as age ≥10 years or age >1 year and age <10 years with WBC ≥50,000/mL; and “infant,” defined as age <1 year). The model with individual-level SES adjustment included the same covariates but additionally adjusted for parental highest education (dichotomized as high school or lower *vs*. some college or more) and annual household income (continuous). Correlations between neighborhood-level and individual-level SES variables were moderate (Pearson coefficients: 0.52–0.64), and both variables were included in the model to address residual confounding (**Supplementary Figure 3**). We assessed the Cox proportional hazards model assumptions analytically by conducting a global likelihood ratio test; the model assumptions were met. Cumulative CES was modeled as a categorical variable on the additive scale. To evaluate potential synergistic effects between pollution and socioeconomic deprivation, we additionally tested for interaction. Additional analyses were performed to evaluate whether other covariates, including year of diagnosis, birthweight, and hospital site, acted as potential confounders. None of these covariates altered the HRs by more than 10% and were therefore excluded from the final model. Stratified analyses were performed by sex, race and ethnicity (non-Latinx White *vs*. Latinx), and cytogenetic characteristic (i.e., deletions in *CDKN2A* and *IKZF1* genes, and high-hyperdiploidy; data were too sparse to analyze *TEL-AML* (*ETV6-RUNX1*) translocation separately with only 3 deaths recorded) to assess for effect modification. All tests were conducted with an alpha level of 0.05. Survival analyses were conducted using R version 4.4.0 in RStudio, with the survival package (version 3.7-0) and the survminer package (version 0.4.9).

## Results

A total of 177 children with leukemia died from non-external causes within five years (124 ALL: 49 AML, and 4 others), mostly attributed to leukemia (n=163), followed by infections (n=4), blood, circulatory, or immune system disorders (n=4), respiratory system disorders (n=2), and other causes (n=4). As expected, children who died were more likely to have AML, high-risk ALL, be diagnosed under the age of one year old, be either Latinx, Asian/Pacific Islanders or Black; and have low parental education and annual household income (**Table 1**). Among the top two tertiles (medium/high) of the composite CES score, the majority were Latinx (65.6%) followed by non-Latinx whites (20.6%) and had lower household income (47.6%) and educational attainment (50%) compared to those in the low composite CES score category (14.2% and 14%, respectively) (**Supplementary Table 1**).

**Table 1:** Characteristics of children with leukemia by survival status at the end of 2020 (1,210 acute lymphoblastic leukemia and 178 acute myeloid leukemia), California Childhood Leukemia Study.

Characteristics	Acute lymphoblastic leukemia		Acute myeloid leukemia	
	Alive (N = 1,086)	Dead (N = 124)	Alive (N = 129)	Dead (N = 49)
Sex				
Female	463 (42.6%)	48 (38.7%)	63 (48.8%)	21 (42.9%)
Male	623 (57.4%)	76 (61.3%)	66 (51.2%)	28 (57.1%)
Race/ethnicity				
Latinx	570 (52.5%)	67 (54.0%)	57 (44.2%)	24 (49.0%)
Non-Latinx White	337 (31.0%)	27 (21.8%)	43 (33.3%)	13 (26.5%)
Non-Latinx Black	26 (2.4%)	8 (6.5%)	4 (3.1%)	2 (4.1%)
Non-Latinx Asian Pacific Islander	86 (7.9%)	12 (9.7%)	11 (8.5%)	6 (12.2%)
Other/Unknown	67 (6.2%)	10 (8.1%)	14 (10.8%)	4 (8.2%)
Birth year				
1982-1989	47 (4.3%)	16 (12.9%)	15 (11.6%)	6 (12.2%)
1990-1999	456 (42%)	65 (52.4%)	57 (44.2%)	18 (36.7%)
2000-2009	458 (42.2%)	34 (27.4%)	43 (33.3%)	21 (42.9%)
2010-2014	125 (11.5%)	9 (7.3%)	14 (10.9%)	4 (8.2%)
Age (years)				
< 1	17 (1.6%)	12 (9.7%)	10 (7.8%)	9 (18.4%)
1 to 2	266 (24.5%)	25 (20.1%)	39 (30.2%)	12 (24.5%)
3 to 6	511 (47.1%)	40 (32.3%)	22 (17.1%)	3(6.1%)
7 to 9	133 (12.2%)	19 (15.3%)	23 (17.8%)	8 (16.3%)
10 to 14	159 (14.6%)	28 (22.6%)	35 (27.1%)	17 (34.7%)
Birth weight (grams)				
< 2500	55 (5.1%)	6 (4.8%)	6 (4.7%)	5 (10.2%)
2500-4000	849 (78.2%)	96 (77.5%)	99 (76.7%)	41 (83.7%)
> 4000	149 (13.7%)	20 (16.1%)	21 (16.3%)	2 (4.1%)
Unknown	33 (3.0%)	2 (1.6%)	3 (2.3%)	1 (2.0%)
Gestational age (weeks)				
< 36	52 (4.8%)	10 (8.1%)	8 (6.2%)	6 (10.2%)
36-41	805 (74.1%)	86 (69.3%)	90 (69.8%)	35 (71.4%)
>41	65 (6.0%)	10 (8.1%)	9 (7.0%)	2 (4.1%)
Missing	164 (15.1%)	18 (14.5%)	22 (17.0%)	7 (14.3%)
NCI risk group for ALL only				
High	275 (25.3%)	44 (35.5%)		
Infant	15 (1.4%)	12 (9.7%)		
Standard	711 (65.6%)	61 (49.2%)		
Unknown	83 (7.7%)	7 (5.6%)		
Household income ($)				
<15,000	183 (16.9%)	24 (19.4%)	31 (24.0%)	6 (12.2%)
15,000 - 29,999	211 (19.4%)	28 (22.6%)	20 (15.5%)	9 (18.4%)
30,000 - 44,999	149 (13.7%)	21 (16.9%)	18 (14.0%)	10 (20.4%)
45,000 - 59,999	145 (13.3%)	20 (16.1%)	12 (9.3%)	9 (18.4%)
60,000 - 74,999	65 (6.0%)	7 (5.6%)	9 (7.0%)	5 (10.2%)
>75,000	333 (30.7%)	24 (19.4%)	39 (30.2%)	10 (20.4%)
Household parental education				
High school or lower	412 (37.9%)	49 (39.5%)	51 (39.5%)	22 (44.9%)
Some college or more	674 (62.1%)	75 (60.5%)	78 (60.5%)	27 (55.1%)

NCI, National Cancer Institute.

### Childhood ALL

**Figure 2:**
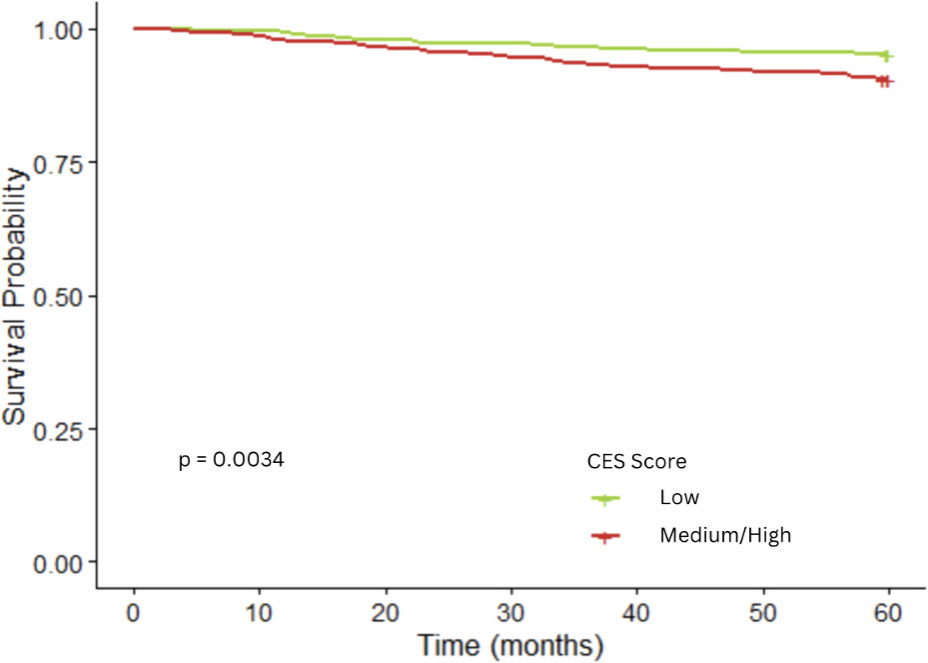
Kaplan–Meier curves for levels of CalEnviroScreen3.0 composite score and childhood acute lymphoblastic leukemia 5-year survival (1,210 children).

Compared to children who survived, a greater proportion of deceased children had their residence at diagnosis classified into the medium/high composite CES score (65% *vs*. 79%, respectively), medium/high population characteristics score (66% *vs*. 77%, respectively), and medium/high pollution burden score (66% *vs*. 75%, respectively) (**Table 2**). Crude Kaplan-Meier analyses showed that the medium/high composite CES score was associated with decreased 5-year survival (p=0.0034; **Figure 2**). Additional Kaplan-Meier analyses showed decreased survival for the CES pollution burden score (p=0.042) (**Supplementary Figure 4**) and population characteristics score (p=0.015) (**Supplementary Figure 5**) in medium/high levels compared to low groups. Notably, survival did not substantially vary by low and medium/high scores for PM2.5 and ozone. The crude cox proportional hazard model showed significant associations for the composite CES (HR = 2.08; 95% CI: 1.26–3.44), population characteristics (HR = 1.80; 95% CI: 1.11–2.92), and pollution burden (HR = 1.62, 95% CI: 1.01–2.58) (**Supplementary Table 2**). In the adjusted models, the risk of death was increased among children with medium/high CES composite scores in comparison to those with low scores in both the partially adjusted Model A (HR = 2.49; 95% CI: 1.42-4.35) and fully adjusted Model B (HR = 2.19; 95% CI: 1.19-3.91) (**Table 2**). Similarly, children with medium/high population characteristic scores were more likely to die (HR in Model A: 1.85; 95% CI: 1.06–3.21); however, the risk estimate decreased, and the confidence interval included the null after further adjusting for individual SES. While pollution burden conferred a 43% increased risk of dying in Model B, the associations were not statistically significant, and no associations were seen with PM2.5 and ozone. To assess whether the association between population characteristics and the outcome was modified by pollution burden, we included an interaction term in the model. The interaction was not statistically significant (p = 0.3), suggesting no evidence of effect modification.

**Table 2:** CalEnviroScreen (CES) 3.0 scores and 5-year survival in 1,210 children treated for acute lymphoblastic leukemia: proportional hazards Cox models without (Model A) and with (Model B) adjustments for individual socioeconomic status.

Characteristics	Alive N = 1,086	Dead N = 124	P-value	Model A^a^			Model B^b^		
				HR	95% CI	P-value	HR	95% CI	P-value
Composite CES score									
Low	375 (35%)	26 (21%)		—	—		—	—	
Medium/high	711 (65%)	98 (79%)	**0.003**	**2.49**	**1.42, 4.35**	**0.001**	**2.19**	**1.19, 3.91**	**0.009**
Population characteristics^c^									
Low	371 (34%)	28 (23%)		—	—		—	—	
Medium/high	715 (66%)	96 (77%)	**0.011**	**1.85**	**1.06, 3.21**	**0.029**	**1.65**	**0.94, 2.93**	0.084
Pollution burden^d^									
Low	368 (34%)	31 (25%)		—	—		—	—	
Medium/high	718 (66%)	93 (75%)	0.053	1.51	0.91, 2.49	0.11	1.43	0.86, 2.37	0.20
PM2.5^d^									
Low	423 (39%)	50 (41%)		—	—		—	—	
Medium/high	661 (61%)	73 (59%)	0.70	1.17	0.76, 1.81	0.50	1.09	0.70, 1.69	0.70
Unknown	2	1							
Ozone^d^									
Low	472 (43%)	54 (44%)		—	—		—	—	
Medium/high	614 (57%)	70 (56%)	>0.90	1.16	0.74, 1.81	0.50	1.09	0.70, 1.71	0.70

a Adjusted for race/ethnicity, birth year, and NCI risk group.

b Adjusted for race/ethnicity, birth year, NCI risk group, household education, and parental income.

c Population characteristics additionally adjusted for pollution burden.

d Pollution burden, PM2.5, and ozone, additionally adjusted for population characteristics.Bolded values represent statistical significance, p-value <0.5.

We evaluated the joint effect of CES scores on 5-year survival of ALL molecular subtypes with favorable *vs*. non-favorable prognosis (high-hyperdiploidy negative. *CDKN2A* and *IKZF1* deletions). In general, children treated for poor prognosis ALL and assigned a medium/high composite CES score exhibited a higher mortality risk compared to other groups (**Supplementary Figure 6**, **Supplementary Tables 3**-**5**). With the exception of ALL with *IKZF1* deletion, these observations appeared to be driven mostly by the population characteristics, PM2.5 burden, and ozone burden, with statistically (or closely) significant HRs equal to 5.11, 2.68, and 2.59, respectively for high-hyperdiploidy negative ALL, and HRs for ALL with *CDKN2A* deletion equal to 3.56 for PM2.5 and 2.52 for ozone. However, formal tests did not reveal statistically significant interactions on multiplicative and additive scales, likely due to limited sample size.

Further adjustment for hospital sites (as a surrogate for access and type of cancer care) did not substantially modify the risk estimates (results not shown). Non-Latinx White children with medium/high composite CES score had a higher risk of death (HR = 3.82, 95% CI: 1.12-13.1), compared to Latinx children (HR = 1.51, 95% CI: 0.59-3.87). The hazard of death associated with medium/high composite CES score was more pronounced in girls (HR = 3.97, 95% CI:1.52-10.4) compared to boys (HR = 1.42, 95% CI: 0.68-3.00). Yet formal tests for interaction (on the multiplicative scale) were not statistically significant.

### Childhood AML

**Figure 3:**
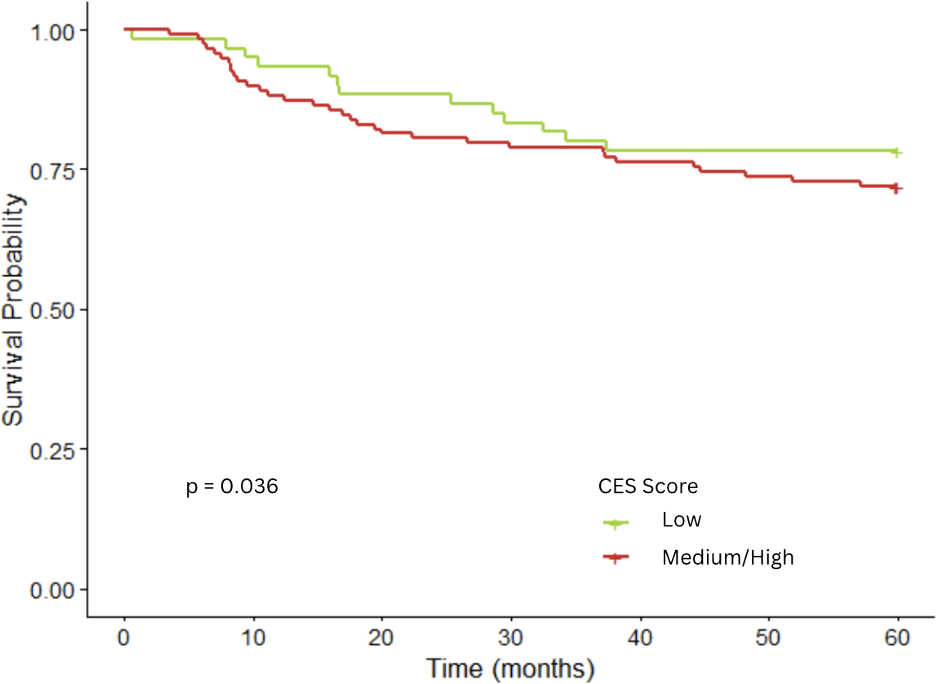
Kaplan–Meier curves for level of CalEnviroScreen3.0 composite score and childhood acute myeloid leukemia 5-year survival (178 children).

The CES composite score was not associated with decreased 5-year survival among children with AML as shown in the Kaplan-Meier analysis (P = 0.36; **Figure 3**), nor was there an association in the crude or fully adjusted proportional hazards models model (**Supplementary Table 6**; **Table 3**). Children with medium/high score for population burden had a two-fold risk of death (HR = 2.21, 95% CI: 0.99–4.94). The other CES scores for overall pollution burden, PM2.5 and ozone were not associated with survival. Sample size, for AML, was too small for meaningful stratified analyses.

**Table 3:** CalEnviroScreen (CES) 3.0 scores and 5-year survival in 178 children treated for acute myeloid leukemia: proportional hazards Cox models, without (Model A) and with (Model B) adjustments for individual socioeconomic status.

Characteristics	Alive N = 129	Dead N = 49	P-value	Model A^a^			Model B^b^		
				HR	95% CI	P-value	HR	95% CI	P-value
Composite CES score									
Low	47 (36%)	13 (27%)		—	—		—	—	
Medium/high	82 (64%)	36 (73%)	**0.003**	1.43	0.71, 2.85	0.3	1.69	0.81, 3.53	0.2
Population characteristics^c^									
Low	48 (37%)	11 (22%)		—	—		—	—	
Medium/high	81 (63%)	38 (78%)	**0.011**	1.88	0.85, 4.14	0.12	2.21	0.99, 4.94	0.053
Pollution burden^d^									
Low	47 (36%)	17 (35%)		—	—		—	—	
Medium/high	82 (64%)	32 (65%)	0.053	1.26	0.65, 2.44	0.5	1.33	0.68, 2.58	0.4
PM2.5^d^									
Low	48 (37%)	21 (43%)		—	—		—	—	
Medium/high	81 (63%)	28 (57%)	0.7	0.99	0.54, 1.84	>0.9	1.03	0.55, 1.90	>0.9
Ozone^d^									
Low	59 (46%)	21 (43%)		—	—		—	—	
Medium/high	70 (54%)	28 (57%)	>0.9	1.42	0.71, 2.82	0.3	1.43	0.73, 2.83	0.3

a Adjusted for race/ethnicity, birth year, and NCI risk group.

b Adjusted for race/ethnicity, birth year, NCI risk group, household education, and parental income.

c Population characteristics additionally adjusted for pollution burden.

d Pollution burden, PM2.5, and ozone, additionally adjusted for population characteristics.Bolded values represent statistical significance, p-value <0.5.

## Discussion

To our knowledge, this study is the first to evaluate environmental pollutants and social drivers of health together in the context of childhood leukemia survival. We found that a medium/high cumulative CES burden based on environmental and social stressors was associated with reduced 5-year survival in children diagnosed with ALL. The medium/high population characteristics score was identified as a major driver of this association. A similar pattern was observed for childhood AML, although it did not reach statistical significance, likely due to small numbers. Medium/high CES scores for PM2.5 and ozone were not associated with decreased survival of childhood ALL overall, but our data suggested increased risks of death for ALL subtypes with unfavorable prognostic.

Our results regarding neighborhood population characteristics are consistent with previous studies showing that lower SES neighborhoods were associated with reduced overall survival among children and young adults in the US and other countries (4, 6, 7, 9). Our study further incorporates adjustments for individual SES factors, such as household income and parental education leading to decrease in HRs, thus highlighting potential residual confounding. This granular individual-level SES data is a major strength of our study compared to registry-based studies that solely relied on neighborhood-level data. Regarding air pollution, a study from Spain found that children and adolescent cancer survivors had lower survival when living in areas with perceived poor air quality (22). A nationwide US study of 172,550 childhood cancer patients using remote-sensing data for PM2.5 found an association between PM2.5 and poorer survival among children with cancer, with effects observed even below the previous annual EPA standard of 12 μg/m³, which has now been lowered to 9 μg/m³ (21); this study, however, did not present leukemia-specific survival data. A study in Utah found a significant association between PM2.5 and all-cause mortality in childhood lymphoid leukemias, even though PM2.5 levels were not associated with leukemia-specific cancer mortality (20). This contrasts with a study in Thailand that examined ambient air pollution, including PM2.5, and found no association with all-cause mortality among children with hematologic malignancies and solid tumors (23). In our study we observed null results for both PM2.5 and ozone with respect to childhood ALL overall. However, subtype analyses further reveal that the impact of environmental and social burdens on survival was more pronounced among children with poor prognostic molecular markers, such as high-hyperdiploidy-negative status, *IKZF1* deletions, and *CDKN2A* deletions. This pattern also held when examining PM2.5 and ozone exposure. Another CCLS study examining early-life exposure to smoking and survival also found results that varied by molecular subtype. Specifically, children with favorable prognoses lost their survival advantage when parental smoking was reported prior to conception (26). This is a different direction than we saw in this study, where children with poorer prognoses experienced worse outcomes following exposure. Underlying mechanisms by which air pollution (and similarly tobacco smoke) affect specific molecular subtypes of leukemia are unclear. It is possible that the body’s response to air pollution affects patients across the cancer continuum from etiology to survival through biological responses such as oxidative stress and inflammatory pathways (32). Alternatively, contaminants in air pollution and tobacco smoke could specifically lead to the development of childhood leukemia subtypes with poor prognosis.

Regarding AML, data were suggestive of a reduced survival among children with medium/high scores for population/social burden, whereas no associations were seen for pollution burden, including individual scores for PM2.5 and ozone. The increase in HRs from the crude model to adjusted Models A and B for AML patients may reflect improved control of residual confounding. However, this pattern was not observed in the ALL analysis, which had a larger sample size, suggesting that the HR estimates for AML may be unstable. No other studies have directly investigated the impact of air pollution and childhood AML survival. It is noteworthy that preconception parental smoking, which shares a mixture of pollutants as seen in air, was found to increase the risk of death from childhood AML in our CCLS cohort (26). Similarly, a study conducted in Texas found that children with AML living near oil or gas wells at the time of diagnosis had an increased risk of mortality, with a dose-response effect linked to closer proximity and a greater number of wells (33). These results with regards to environmental pollutants and AML survival highlight the need for further research with larger samples, especially at different life stages.

A growing body of evidence suggests that environmental exposures, particularly air pollution, may negatively influence survival outcomes in pediatric cancer. Epidemiologic studies from Spain, Utah, and the national US have identified associations between exposure to PM2.5 and increased mortality risk among children with cancer (20–22). Several biological mechanisms underlying these associations may be considered. Air pollution has been implicated in immune dysregulation through multiple pathways. Inhaled pollutants, such as PM2.5, induce systemic inflammation and oxidative stress, activating proinflammatory cytokines and signaling cascades that promote immune cell activation and chronic inflammation (34–36). These immune alterations can impair surveillance and regulatory functions, creating a permissive environment for disease progression, including malignancy (37). Pollutants have also been shown to disrupt the epigenetic-immune axis, leading to lasting immune dysfunction (38, 39). Specifically, air pollution can alter DNA methylation and other epigenetic markers, contributing to dysregulated gene expression, sustained inflammatory responses, and increased vulnerability to autoimmune diseases, chronic infections, and cancer (40). Together, these mechanisms provide a plausible biological basis for the poorer survival we observed among children facing higher environmental and social burdens. Despite seeing no association between PM2.5 levels and survival in ALL overall, our data suggested that some ALL subtypes may be more sensitive than others, supporting the role of environmental factors modulating immune pathways that could underline subtype-specific risk and survival differences.

Stratified analyses by race and ethnicity suggested that Latinx children, despite comprising the majority of those in the medium/high CES tertiles, had an attenuated risk compared to non-Latinx White children. However, a formal test for statistical interaction on the multiplicative scale was not significant, limiting our ability to interpret the results in our study. Within a larger context of children’s health, studies have documented that children from socially adverse backgrounds have resiliencies for outcomes across their lives, such as cultural identity, community cohesion, and relational and school factors (41). It is possible that cultural or physical built factors that mitigate the adverse effect of environmental and social stressors through areas of resilience may explain our unexpected observation.

A key strength of the study is the availability of clinical data including prognostic risk classification and molecular subtypes. These detailed data enhance the precision of our analyses and provide valuable insights into disease heterogeneity, contributing to a more nuanced understanding of childhood leukemia outcomes. An additional strength of the study is the inclusion of individual-level SES data obtained through parental interviews, i.e., parental education and household education, etc., which increases the robustness of our analyses. Although models were adjusted for both neighborhood and individual-level SES, there may still be some residual confounding by SES. Previous research has highlighted that medical insurance and access to supportive care may differ by geographic location and socio-demographic situations, leading to differential adverse outcomes (42). However, adjustment for health insurance and hospital study site (as a proxy for treatment modality and supportive care) did not affect the relationship between SES and leukemia survival, suggesting that access to care should not substantially impact our results.

Limitations of our study include potential misclassification and spatial uncertainties in the CES 3.0 exposure data, which may arise from data accuracy issues, source limitations, and the fact that these tools are not formal risk assessments (43). Additionally, some temporal misalignment exists between participants’ enrollment period (1995–2015) and the timing of CES 3.0 exposure data collection (e.g., 2012–2014 for PM2.5). To account for this, the models were adjusted for birth year to account for calendar trends, and date of diagnosis which did not substantially alter the HRs. Despite potential exposure misclassification due to temporal misalignment, it should be noted that historical disparities in air pollution exposure have remained persistent over time. Studies in the US and California show that while air pollution levels, including PM2.5, have improved over time, communities of color continue to experience disproportionately higher exposure rates (44, 45). Furthermore, because CES is percentile-scaled based on the maximum exposure levels within the dataset, communities ranked in the highest exposure percentiles today were also likely in at least the medium to high exposure categories in previous years. Another limitation of this study is its geographic specificity; the findings are based on data from California and may not be generalizable to other regions with differing environmental conditions, pollution levels, or socioeconomic contexts. In our study, parental interviews were not completed for 50 children who died shortly after enrollment, raising issue of potential survival bias. However, the 5-year survival rate for ALL combined was 91.8%, and for AML, it was 69.9%, consistent with national data for our study period (1995–2015) (46, 47). Deceased children without interviews were similar to deceased children with interviews in terms of birth registry data and sociodemographic characteristics, but there was a suggestion that neighborhood income differed between households that were not interviewed and those that were (mean = $38,000 *vs*. $45,300, respectively; pooled t-test, P = 0.06), so that some level of selection bias cannot be ruled out. While we observed subtype-specific associations, these analyses were based on small subgroups, which may have limited statistical power. Thus, findings should be interpreted with caution and considered exploratory. Larger studies are needed to validate these preliminary observations. Detailed information on treatment that was administered (i.e., types, doses, and timing) was not available in our dataset, which limited our ability to examine their influence on survival outcomes. To mitigate this limitation, we have accounted for NCI risk groups for childhood ALL, as a surrogate for treatment regimens. Based on previous findings that absence of high-hyperdiploidy and *CDKN2A* or *IKZF1* deletions are factors for an unfavorable prognosis (31), we examined these molecular subtypes using our available data. Yet, information for other prognostic subtypes, such as Ph+ and Ph-like ALL or those with the *TEL-AML* (*ETV6-RUNX1*) fusion gene, was not available or lacked sufficient sample size. Lastly, analysis of rare subtypes and racial and ethnic minority groups was based on small numbers.

In conclusion, our data suggest that a higher cumulative burden from environmental contaminants and social deprivation was associated with reduced survival of childhood ALL and AML, in a California-based population. The score for population characteristics was the main driver of these observations for ALL, and also AML. Medium/high scores for PM2.5 and ozone were possibly associated with reduced survival of specific ALL subtypes, adding to the body of evidence suggesting a harmful effect of chemicals on leukemia survival. These results emphasize the need for further investigation into the interplay between environmental exposures, social determinants, and leukemia prognosis, and to integrate this information into treatment plans and survivorship care. Future studies would be strengthened by having direct and individual measures of pollutant exposures, and by incorporating data on treatment regimens to better inform personalized approaches to care. As more data support the deleterious roles of environmental and social burden on the cancer continuum from etiology to clinical outcomes, it is important to consider primary, secondary, and tertiary prevention strategies for cancer patients at the individual and policy levels to further reduce health disparities.

## Data Availability

The data analyzed in this study is subject to the following licenses/restrictions: The CES 3.0 exposure data used in this study are publicly available through the California Office of Environmental Health Hazard Assessment. The epidemiologic and clinical data from the California Childhood Leukemia Study are not publicly available due to the terms of informed consent signed by participants at enrollment but are available upon reasonable request to CM, cmetayer@berkeley.edu. Mortality data analyzed in this study were obtained from the California Department of Public Health (CDPH) Center for Health Statistics and Informatics (CHSI) and are not publicly available due to the terms outlined by CDPH-CHSI. Requests to access data should be directed to CDPH-CHSI, CHSIVitalRecords@cdph.ca.gov.
