# Supplementary material for: The impact of environmental contaminants, air pollution, and social deprivation on childhood leukemia survival in California
Source: Front Oncol. 2025 Dec 8;15:1686115. doi: 10.3389/fonc.2025.1686115 (PMC12719266; doi:10.3389/fonc.2025.1686115)
Supplement: Supplementary file 1 [file DataSheet1.docx]

**Supplemental Tables/Figures:**

**Supplemental Table 1.** California Childhood Leukemia SES data by CalEnviroScreen (CES) 3.0 composite exposure classification

| **Characteristic** | **Low**  **N = 471** | **Medium/High**  **N = 943** |
| --- | --- | --- |
| **Leukemia Type** |  |  |
| ALL | 401 (85.0%) | 809 (85.8%) |
| AML | 60 (13.0%) | 118 (12.5%) |
| Other | 10 (2.0%) | 16 (1.7%) |
| **Sex** |  |  |
| Female | 198 (42.0%) | 407 (43.0%) |
| Male | 273 (58.0%) | 536 (57.0%) |
| **Race/Ethnicity** |  |  |
| Latinx | 109 (23.1%) | 619 (65.6%) |
| Non-Latinx White | 237 (50.3%) | 194 (20.6%) |
| Non-Latinx Black | 7 (1.5%) | 33 (3.5%) |
| Non-Latinx Asian/Pacific  Islander | 71(15.1%) | 46 (4.9%) |
| Other/Unknown | 47 (10.0%) | 51 (5.4%) |
| **Household income ($)** |  |  |
| <15,000 | 31 (6.6%) | 216 (22.9%) |
| 15,000 - 29,999 | 36 (7.6%) | 233 (24.7%) |
| 30,000 - 44,999 | 43 (9.1%) | 159 (16.9%) |
| 45,000 - 59,999 | 60 (12.7%) | 131 (13.9%) |
| 60,000 - 74,999 | 33 (7.0%) | 56 (5.9%) |
| 75,000 + | 268 (57.0%) | 148 (15.7%) |
| **Household parental education** |  |  |
| High school or lower | 65 (14.0%) | 475 (50.0%) |
| Some college or more | 406 (86.0%) | 468 (50.0%) |

**Supplemental Table 2.** CalEnviroScreen (CES) 3.0 scores and 5-year survival in 1,210 children treated for acute lymphoblastic leukemia: crude proportional hazards Cox model.

| **Characteristic** | **HR** | **95% CI** | **p-value** |
| --- | --- | --- | --- |
| **Composite CES score** |  |  |  |
| Low |  |  |  |
| Medium/high | **2.08** | **1.26, 3.44** | **0.004** |
| **Population Characteristics** |  |  |  |
| Low |  |  |  |
| Medium/high | **1.8** | **1.11, 2.92** | **0.017** |
| **Pollution Burden** |  |  |  |
| Low |  |  |  |
| Medium/high | **1.62** | **1.01, 2.58** | **0.044** |
| **PM2.5** |  |  |  |
| Low |  |  |  |
| Medium/high | 1.08 | 0.71, 1.63 | 0.7 |
| **Ozone** |  |  |  |
| Low |  |  |  |
| Medium/high | 1.15 | 0.77, 1.73 | 0.5 |

**Supplemental Table 3.** Joint effects of CalEnviroScreen (CES) 3.0 scores and molecular type of childhood acute lymphoblastic leukemia (favorable vs. poor prognosis) on survival: proportional hazards Cox models.

| **Characteristic** | **Alive**  **n (%)** | **Dead**  **n (%)** | **HR^a^** | **95% CI** | **p-value** |
| --- | --- | --- | --- | --- | --- |
| **High-hyperdiploidy/composite CES** |  |  |  |  |  |
| Yes (favorable) / Non-exposed | 99 (11%) | 4 (4%) | 1 (ref) |  |  |
| Yes (favorable) / Exposed | 192 (22%) | 14 (13.5%) | 1.65 | 0.44, 6.16 | 0.46 |
| No (poor) / Non-exposed | 224 (26%) | 17 (16.5%) | 1.4 | 0.39, 5.05 | 0.61 |
| No (poor) / Exposed | 354 (41%) | 68 (66%) | **4.01** | **1.22, 13.2** | **0.02** |
| **High-hyperdiploidy/Population characteristics^b^** |  |  |  |  |  |
| Yes (favorable) / Non-exposed | 100 (11.5%) | 3 (3%) | 1 (ref) |  |  |
| Yes (favorable) / Exposed | 191 (22%) | 15 (14.5%) | 2.27 | 0.49, 10.50 | 0.29 |
| No (poor) / Non-exposed | 217 (25%) | 17 (16.5%) | 2.04 | 0.45, 9.27 | 0.36 |
| No (poor) / Exposed | 361 (41.5%) | 68 (66%) | **5.11** | **1.22, 21.44** | **0.03** |
| **High-hyperdiploidy/Pollution burden^c^** |  |  |  |  |  |
| Yes (favorable) / Non-exposed | 86 (10%) | 5 (5%) | 1 (ref) |  |  |
| Yes (favorable) / Exposed | 205 (23.5%) | 14 (13% | 1.19 | 0.32, 4.41 | 0.80 |
| No (poor) / Non-exposed | 216 (25%) | 22 (21%) | 2.07 | 0.60, 7.13 | 0.25 |
| No (poor) / Exposed | 362 (41.5%) | 63 (61%) | 2.70 | 0.82, 8.81 | 0.10 |
| **High-hyperdiploidy/PM2.5^c^** |  |  |  |  |  |
| Yes (favorable) / Non-exposed | 108 (12%) | 10 (10%) | 1 (ref) |  |  |
| Yes (favorable) / Exposed | 183 (21%) | 8 (8%) | 1.23 | 0.39, 3.93 | 0.72 |
| No (poor) / Non-exposed | 251 (29%) | 33 (32%) | 2.20 | 0.83, 5.84 | 0.11 |
| No (poor) / Exposed | 326 (38%) | 52 (50%) | **2.68** | **1.03 6.96** | **0.04** |
| **High-hyperdiploidy/ozone^c^** |  |  |  |  |  |
| Yes (favorable) / Non-exposed | 122 (14%) | 9 (8.5%) | 1 (ref) |  |  |
| Yes (favorable) / Exposed | 169 (19.5%) | 9 (8.5%) | 1.09 | 0.34, 3.48 | 0.88 |
| No (poor) / Non-exposed | 287 (33%) | 38 (37%) | 2.02 | 0.77, 5.30 | 0.15 |
| No (poor) / Exposed | 291 (33.5%) | 47 (46%) | 2.59 | 0.98, 6.84 | 0.054 |

* Exposed = medium/high category; Non-exposed = low category

a Adjusted for race/ethnicity, birth year, NCI risk group, household education, and parental

income

b Population characteristics additionally adjusted for pollution burden

c Pollution burden, PM2.5, and ozone additionally adjusted for population characteristics

**Supplemental Table 4.** Joint effects of CalEnviroScreen (CES) 3.0 scores and molecular type of childhood acute lymphoblastic leukemia (favorable vs. poor prognosis) on survival: proportional hazards Cox models.

| **Characteristic** | **Alive**  **n (%)** | **Dead**  **n (%)** | **HR^a^** | **95% CI** | **p-value** |
| --- | --- | --- | --- | --- | --- |
| ***CDKN2A* deletion/ composite CES** |  |  |  |  |  |
| No (favorable) / Non-exposed | 125 (23.8%) | 5 (8.2%) | 1 (ref) |  |  |
| No (favorable) / Exposed | 259 (49.3%) | 31 (51.0%) | 2.45 | 0.81, 7.43 | 0.11 |
| Yes (poor) / Non-exposed | 41 (7.8%) | 6 (9.8%) | 2.88 | 0.76, 10.9 | 0.12 |
| Yes (poor) / Exposed | 100 (19.1%) | 19 (31.0%) | **3.91** | **1.21, 12.6** | **0.022** |
| ***CDKN2A* deletion/Population Characteristics^b^** |  |  |  |  |  |
| No (favorable) / Non-exposed | 121 (23.0%) | 6 (9.8%) | 1 (ref) |  |  |
| No (favorable) / Exposed | 263 (50.1%) | 30 (49.2%) | 1.55 | 0.55, 4.0 | 0.4 |
| Yes (poor) / Non-exposed | 44 (8.4%) | 7 (11.0%) | 2.67 | 0.79, 9.06 | 0.11 |
| Yes (poor) / Exposed | 97 (18.5%) | 18 (30.0%) | 2.52 | 0.85, 7.50 | 0.10 |
| ***CDKN2A* deletion/Pollution Burden^c^** |  |  |  |  |  |
| No (favorable) / Non-exposed | 124 (23.6%) | 7 (11.0%) | 1 (ref) |  |  |
| No (favorable) / Exposed | 260 (49.6%) | 29 (48.0%) | 1.88 | 0.70, 5.08 | 0.20 |
| Yes (poor) / Non-exposed | 50 (9.5%) | 9 (15.0%) | **3.15** | **1.01, 9.86** | **0.048** |
| Yes (poor) / Exposed | 91 (17.3%) | 16 (26.0%) | 2.79 | 0.95, 8.15 | 0.061 |
| ***CDKN2A* deletion/PM2.5^c^** |  |  |  |  |  |
| No (favorable) / Non-exposed | 165 (31.4%) | 11 (18.0%) | 1 (ref) |  |  |
| No (favorable) / Exposed | 219 (42.0%) | 25 (41.0%) | **2.54** | **1.06, 6.10** | **0.037** |
| Yes (poor) / Non-exposed | 45 (8.6%) | 10 (16.0%) | 2.91 | 0.98, 8.65 | 0.055 |
| Yes (poor) / Exposed | 95 (18.0%) | 15 (25.0%) | **3.56** | **1.39, 9.10** | **0.008** |
| ***CDKN2A* deletion/Ozone^c^** |  |  |  |  |  |
| No (favorable) / Non-exposed | 186 (35.0%) | 14 (23.0%) | 1 (ref) |  |  |
| No (favorable) / Exposed | 198 (38.0%) | 22 (36.0%) | 1.57 | 0.71, 3.47 | 0.30 |
| Yes (poor) / Non-exposed | 57 (11.0%) | 11 (18.0%) | 2.01 | 0.8, 5.04 | 0.14 |
| Yes (poor) / Exposed | 84 (16.0%) | 14 (23.0%) | **2.52** | **1.06, 6.00** | **0.037** |

* Exposed = medium/high category; Non-exposed = low category

a Adjusted for race/ethnicity, birth year, NCI risk group, household education, and parental

income

b Population characteristics additionally adjusted for pollution burden

c Pollution burden, PM2.5, and ozone, additionally adjusted for population characteristics

**Supplemental Table 5.** Joint effects of CalEnviroScreen (CES) 3.0 scores and molecular type of childhood acute lymphoblastic leukemia (favorable vs. poor prognosis) on survival: proportional hazards Cox models.

| **Characteristic** | **Alive**  **n (%)** | **Dead**  **n(%)** | **HR^a^** | **95% CI** | **p-value** |
| --- | --- | --- | --- | --- | --- |
| ***IKZF1* deletion/ composite CES** |  |  |  |  |  |
| No (favorable) / Non-exposed | 148 (28.2%) | 10 (17.0%) | 1 (ref) |  |  |
| No (favorable) / Exposed | 308 (58.7%) | 33 (55.9%) | 1.45 | 0.63, 3.34 | 0.40 |
| Yes (poor) / Non-exposed | 18 (3.4%) | 1 (1.7%) | 0 | 0.00, Inf | >0.9 |
| Yes (poor) / Exposed | 51 (9.7%) | 15 (25.4%) | **2.88** | **1.05, 7.89** | **0.04** |
| ***IKZF1* deletion/Population Characteristics^b^** |  |  |  |  |  |
| No (favorable) / Non-exposed | 149 (28.4%) | 12 (20.3%) | 1 (ref) |  |  |
| No (favorable) / Exposed | 307 (58.5%) | 31 (52.6) | 0.99 | 0.45, 2.19 | >0.9 |
| Yes (poor) / Non-exposed | 16 (3.0%) | 1 (1.7%) | 0 | 0.00, Inf | >0.9 |
| Yes (poor) / Exposed | 53 (10.1%) | 15 (25.4%) | 1.92 | 0.73, 5.04 | 0.20 |
| ***IKZF1* deletion/Pollution Burden^c^** |  |  |  |  |  |
| No (favorable) / Non-exposed | 154 (29.3%) | 12 (20.3%) | 1 (ref) |  |  |
| No (favorable) / Exposed | 302 (57.5%) | 31 (52.6%) | 1.32 | 0.61, 2.84 | 0.50 |
| Yes (poor) / Non-exposed | 20 (3.8%) | 4 (6.8%) | 2 | 0.54, 7.46 | 0.30 |
| Yes (poor) / Exposed | 49 (9.4%) | 12 (20.3%) | 1.87 | 0.68, 5.12 | 0.20 |
| ***IKZF1* deletion/PM2.5^c^** |  |  |  |  |  |
| No (favorable) / Non-exposed | 185 (35.3%) | 13 (22.0%) | 1 (ref) |  |  |
| No (favorable) / Exposed | 270 (51.4%) | 30 (50.8%) | 2.11 | 0.98, 4.53 | 0.056 |
| Yes (poor) / Non-exposed | 25 (4.8%) | 8 (13.6%) | 2.33 | 0.70, 7.78 | 0.20 |
| Yes (poor) / Exposed | 44 (8.5%) | 8 (13.6%) | 2.64 | 0.96, 7.21 | 0.059 |
| ***IKZF1* deletion/Ozone^c^** |  |  |  |  |  |
| No (favorable) / Non-exposed | 219 (41.7%) | 19 (32.0%) | 1 (ref) |  |  |
| No (favorable) / Exposed | 237 (45.1%) | 24 (41.0%) | 1.36 | 0.67, 2.78 | 0.40 |
| Yes (poor) / Non-exposed | 24 (4.6%) | 6 (10.0%) | 1.62 | 0.52, 5.06 | 0.40 |
| Yes (poor) / Exposed | 45 (8.6%) | 10 (17.0%) | 1.98 | 0.76, 5.17 | 0.20 |

* Exposed = medium/high category; Non-exposed = low category

a Adjusted for race/ethnicity, birth year, NCI risk group, household education, and parental

income

b Population characteristics additionally adjusted for pollution burden

c Pollution burden, PM2.5, and ozone, additionally adjusted for population characteristics

**Supplemental Table 6.** CalEnviroScreen (CES) 3.0 scores and 5-year survival in 178 children treated for acute myeloid leukemia: crude proportional hazards Cox model.

| **Characteristic** | **HR** | **95% CI** | **p-value** |
| --- | --- | --- | --- |
| **Composite CES score** |  |  |  |
| Low |  |  |  |
| Medium/high | 1.35 | 0.71, 2.56 | 0.4 |
| **Population Characteristics** |  |  |  |
| Low |  |  |  |
| Medium/high | 1.7 | 0.86, 3.34 | 0.13 |
| **Pollution Burden** |  |  |  |
| Low |  |  |  |
| Medium/high | 1.14 | 0.62, 2.12 | 0.7 |
| **PM2.5** |  |  |  |
| Low |  |  |  |
| Medium/high | 0.9 | 0.50, 1.62 | 0.7 |
| **Ozone** |  |  |  |
| Low |  |  |  |
| Medium/high | 1.33 | 0.74, 2.41 | 0.3 |


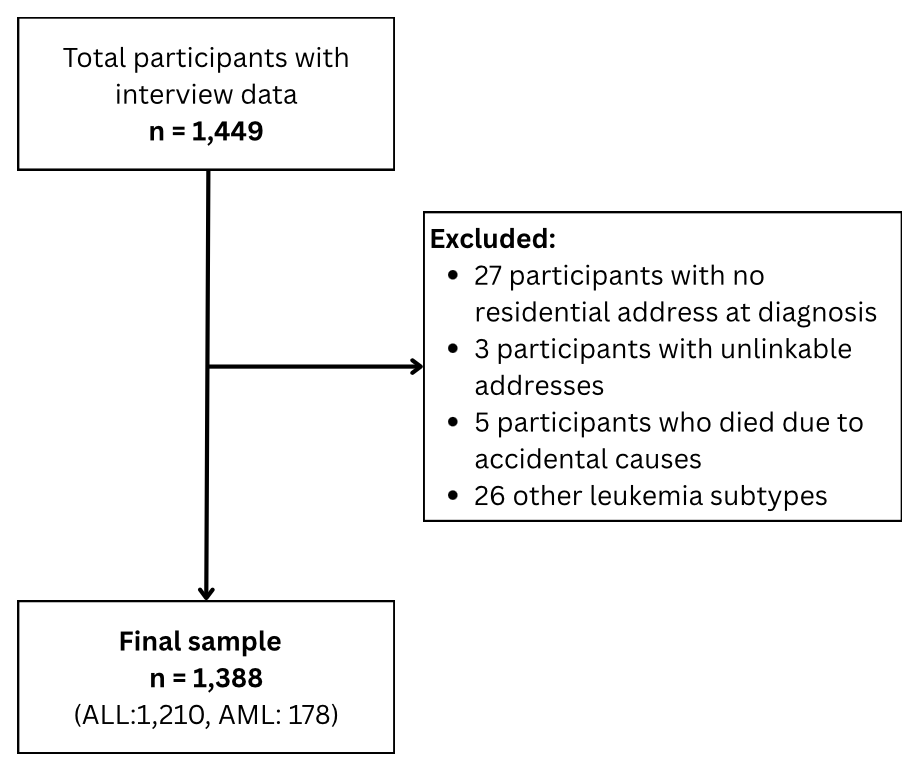
**Supplemental Figure 1.** Inclusion and exclusion of participants in the study


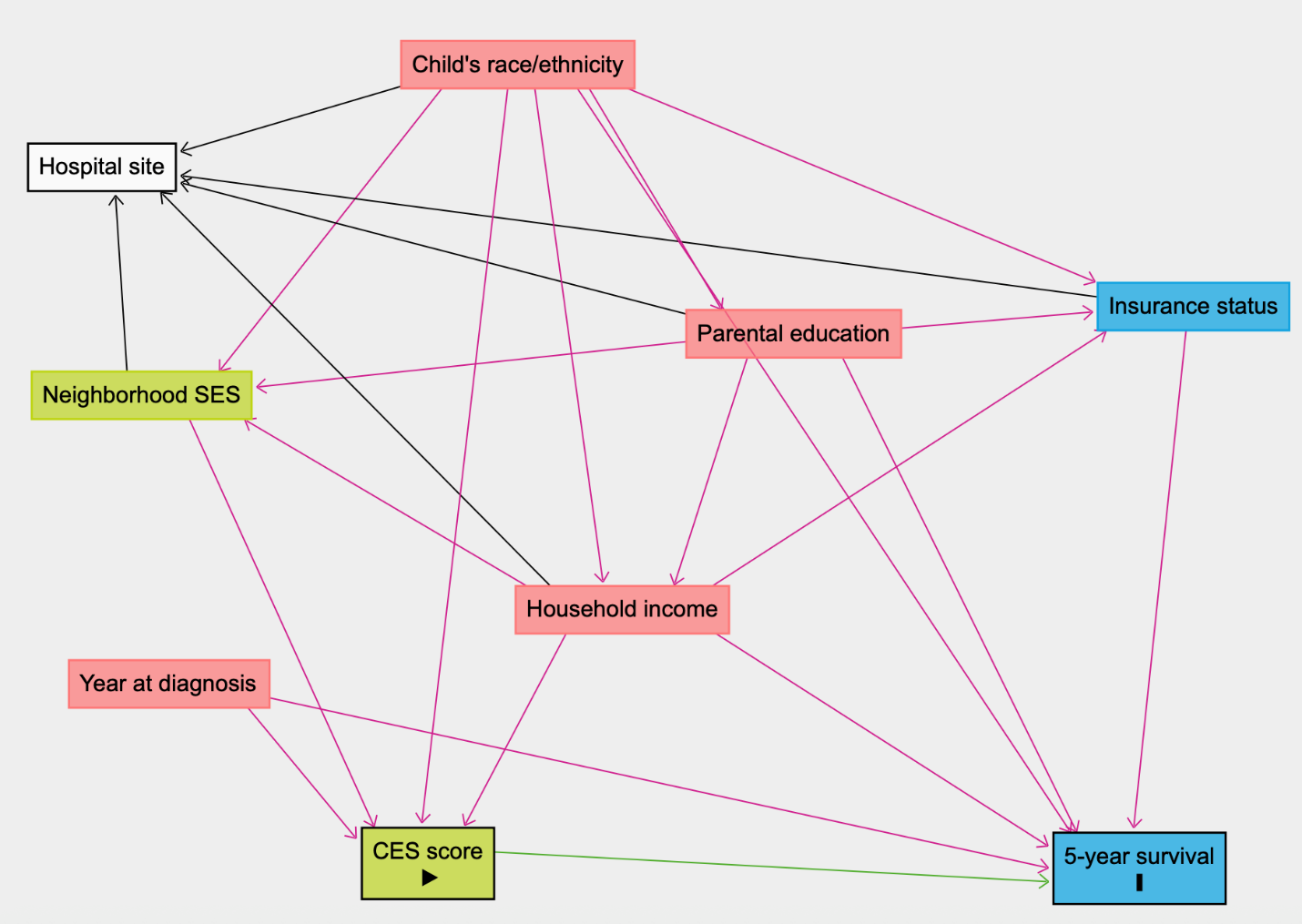
**Supplemental Figure 2**. Directed Acyclic Graph (DAG)


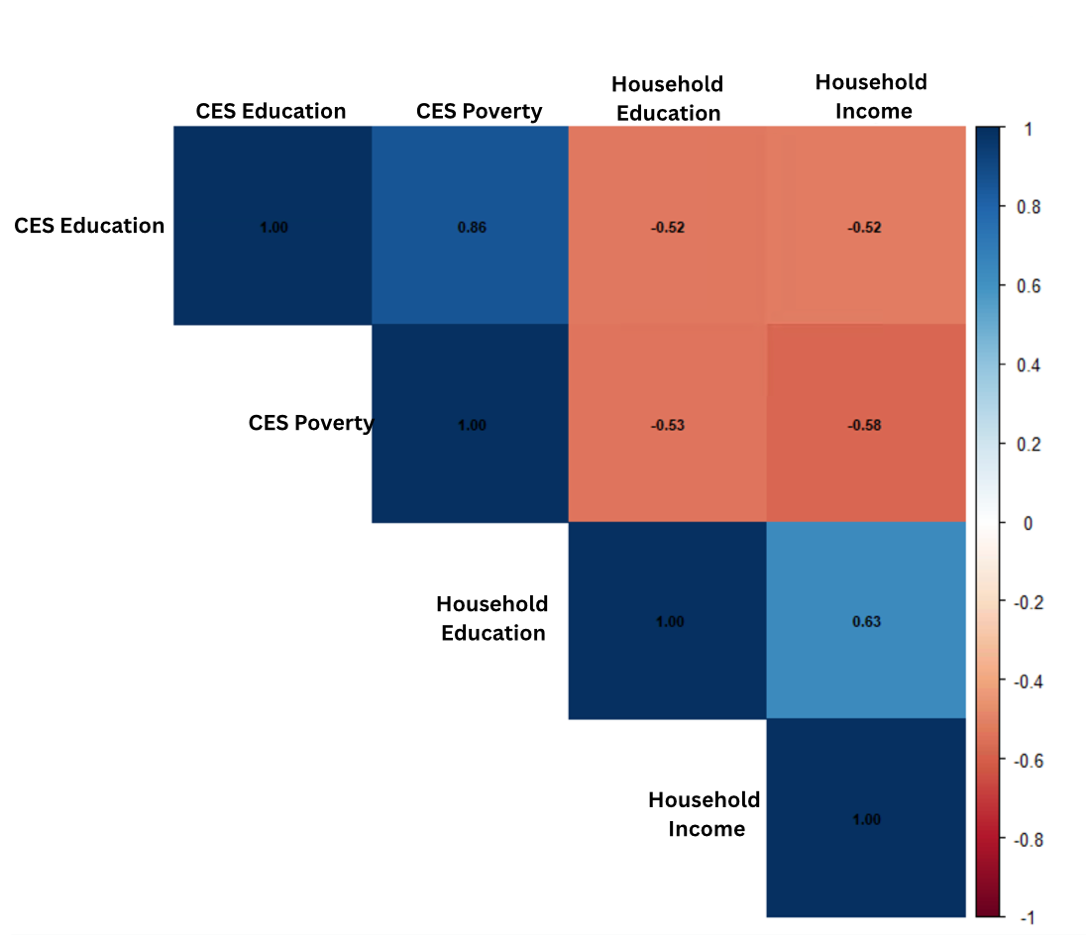
**Supplemental Figure 3**. Correlation Matrix: Individual-Level Household Socioeconomic Conditions vs. CalEnviroScreen 3.0 Census Tract Socioeconomic Indicators

**Supplemental Figure 4**. Kaplan–Meier curves for levels of CalEnviroScreen3.0 pollution burden score and childhood acute lymphoblastic leukemia 5-year survival (1,210 children).


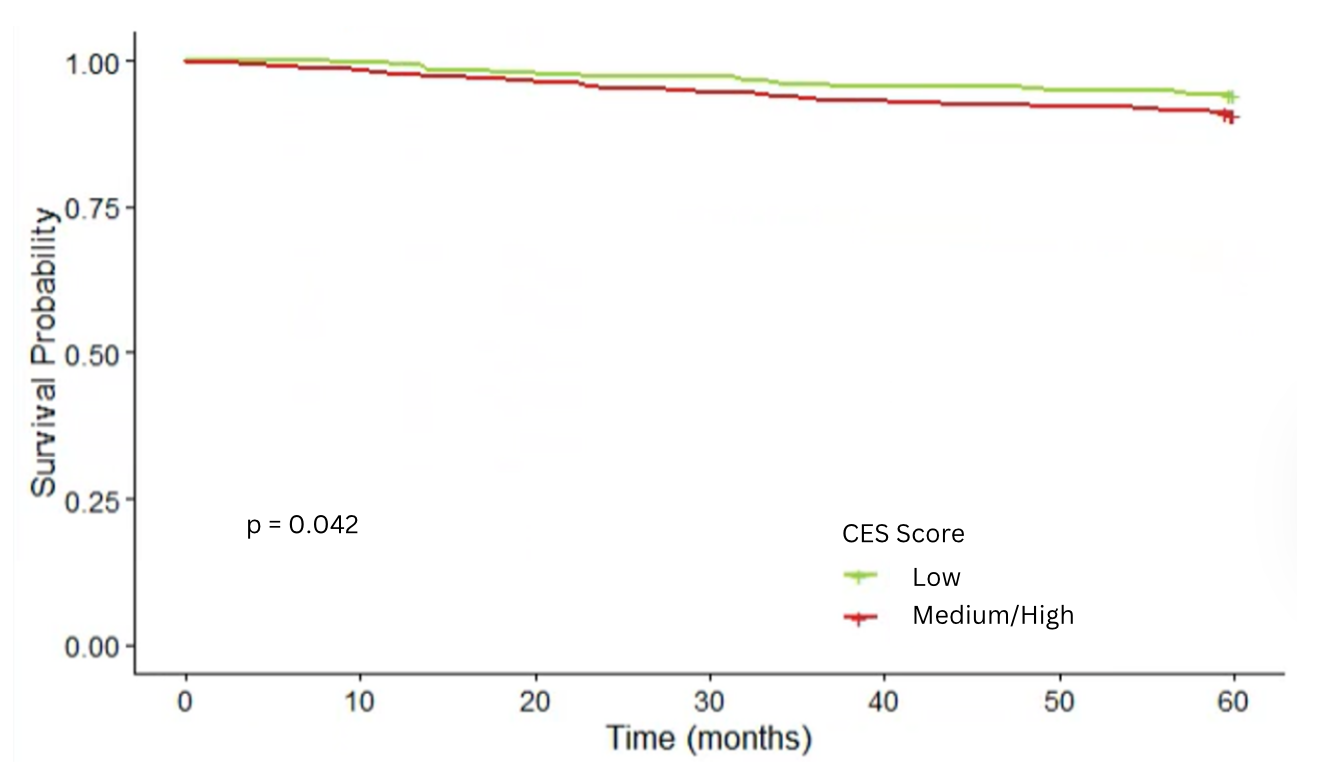


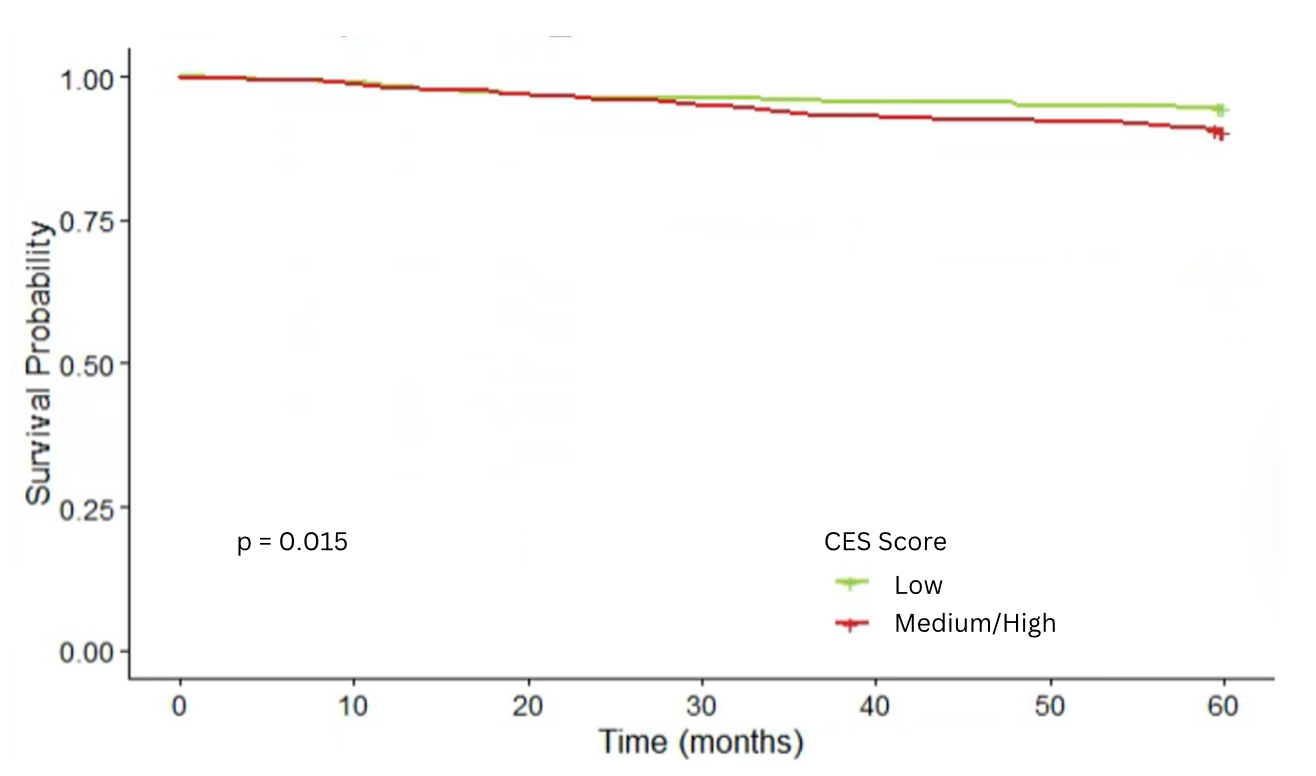
**Supplemental Figure 5.** Kaplan–Meier curves for levels of CalEnviroScreen3.0 population characteristics score and childhood acute lymphoblastic leukemia 5-year survival (1,210 children).


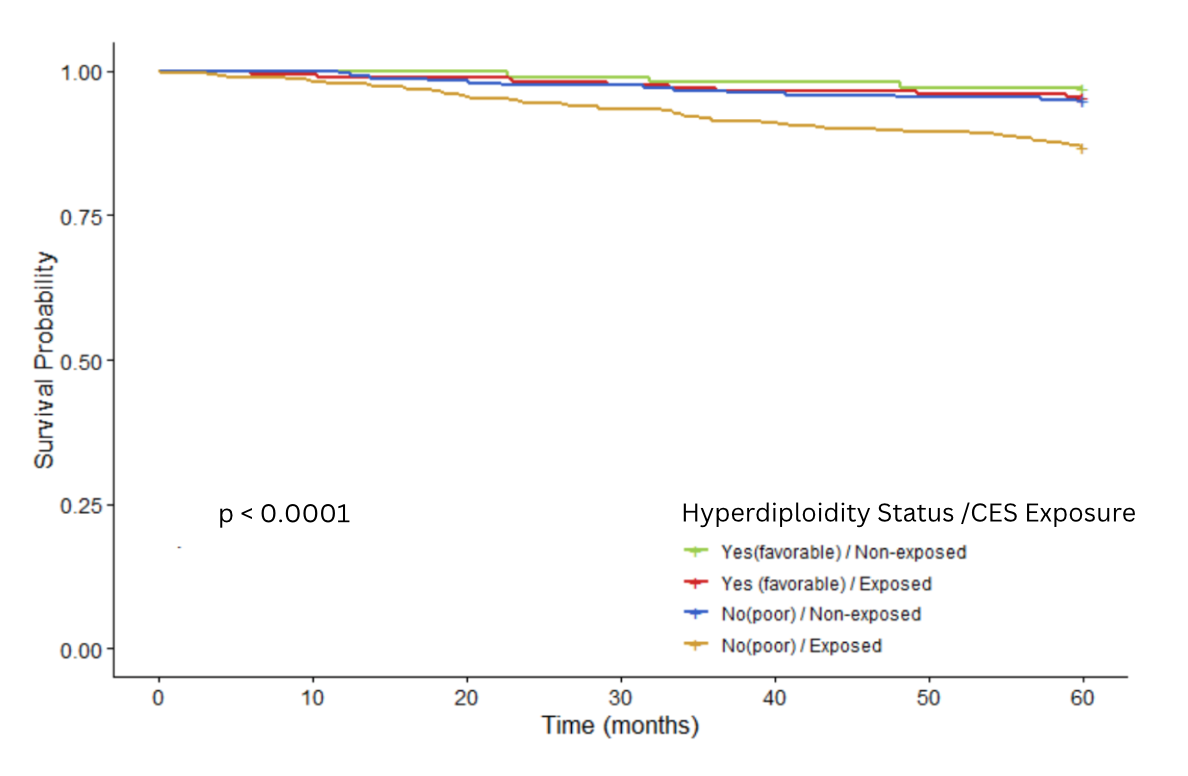
**Supplemental Figure 6.** Kaplan–Meier curves for joint effects of CalEnviroScreen3.0 composite score and hyperdiploidy status on childhood acute lymphoblastic leukemia 5-year survival (972 children).
